# Navigating Amazonia under uncertainty: past, present and future environmental governance

**DOI:** 10.1098/rstb.2007.0023

**Published:** 2008-02-11

**Authors:** Emily Boyd

**Affiliations:** Centre for the Environment, Environmental Change Institute, Oxford UniversitySouth Parks Road, Oxford OX1 3YB, UK

**Keywords:** dieback, governance, adaptive

## Abstract

One of the major environmental challenges of the twenty-first century is the continued rapid deforestation of Amazonia. The 2005 dieback crisis emphasizes the unprecedented challenges facing Brazil. The examination of past and present institutions for ecosystem management, in Amazonia, shows structural barriers across public, private and community arrangements. The adaptive governance concept helps to understand why these institutions are failing to deliver sustainable futures. In looking forward, it is encouraging to see that important networks of knowledge and a number of novel initiatives are emerging in Brazil. These new arrangements are novel in the way that they seem to be adaptive and navigate structures in the hope of overcoming insurmountable drivers of deforestation.

## 1. Introduction

In 2005, Brazil experienced one of the worst droughts in 30 years compounded by extensive forest fires. The cause appears to have been warmer global temperatures, which led to hotter ocean surface temperatures and ultimately lower rainfall across several regions of the country. The drought impacted the northeast, as well as southwest and western Amazonia. A state of emergency was called and the Brazilian government mobilized its army to provide water and medical supplies to isolated communities and contend with the intense forest fires in Brazil's western state of Acre (http://news.bbc.co.uk/1/hi/world/americas/4272116.stm). The resulting smoke pollution affected more than 400 000 people, and the fire damaged more than 300 000 ha of rainforest; direct costs amounted to more than US$50 million (Brown *et al.* 2006). The true monetary and health costs could be far higher as the widespread damage caused to forest cover has made the area more susceptible to repeated burning.

Perhaps it was the scale of the unfolding crisis that instigated what is arguably a novel and successful response. Critical to the disaster response was the availability of satellite imagery, hot spot data and meteorological data, which first convinced the Governor of Acre to act by prohibiting fires. Brown *et al.* (2006) explain the details of the response process. To summarize, near-real-time data on hot spot distributions, derived from MODIS (moderate resolution imaging spectroradiometer) images and custom-designed analysis software, were voluntarily made available to state government officials by a team of NASA-supported scientists working on the large-scale biosphere–atmosphere experiment in Amazonia (http://lba.cptec.inpe.br/lba/site/). The Acre government in turn established a ‘situation room’ staffed by two civil defence coordinators, three state employees from INPE and several researchers and students from the LBA-ECO team. Using both satellite imagery and on-the-ground information, the team provided daily briefings by email on the locations of fires to the local authorities and the Brazilian army, helping to coordinate and focus state and national efforts. Following the successful response to the crisis, access to CBERS-2 satellite imagery is now granted to Brazilian institutions and more widely across South America. The Environmental Institute of Acre has also since established a permanent situation room that incorporates the use of multiple satellite sensors to monitor the extent of fire and drought conditions (Berkes & Seixas 2004).

What is particularly important about the 2005 Amazonian dieback was the speed and magnitude of the events. An important insight is that ecological systems do not respond to stress such as high temperatures or extreme weather events in a linear or predictable manner. In fact, even small disturbances can bring about large and sometimes irreversible changes (e.g. dieback). The governance system that tackled the 2005 crises was unconventional in its rapid response and in the establishment of a situation room, extensive networks and reliance on available information on the internet. One may wonder whether such a ‘flexible’ governance system can be institutionalized, strengthened or replicated to cope with the future climate-related surprises in Amazonia.

Still, it is evident that the existing governance and management strategies for Amazonia have largely fallen short of adequately protecting both people and ecosystems. To blame are both global economic demand for raw materials, minerals and agricultural commodities and weak enforcement of policies at the national level. In Brazil, federal command and control structures have failed to deliver conservation (Fearnside 2005, 2006), and state-level administration has failed to enforce law relating to forests and land-use change or to provide incentives to reduce deforestation (Chomitz *et al.* 2006). Given these potentially insurmountable constraints, new forms of governance are emerging, which navigate the barriers to sustainable futures via networks. One emerging framework developed from the observation of several hundred cases of ecosystems management over the past 20 years is ‘adaptive governance’ that emphasizes complexity, rather than the steady-state equilibrium, as a pre-determinant of successful environmental governance. Adaptive governance consists of four fundamentals: explicit understanding of the ecosystem; monitoring; flexibility in management and administration through networks; and preparation for ‘surprise’. In the Amazon, the notion of adaptive governance may appear impossibly far from the historical and current reality of land management. However, the framework provides direction and a benchmark for future efforts to improve governance for the region's long-term protection. While it is important to have governance systems that are able to respond quickly to surprise in the system, it is also necessary to have the structures in place to prevent the occurrence of such disasters in the first place. Any attempt to manage Amazonia will require a suite of approaches and mechanisms, such as local innovations and scientific research, coupled with national regulation and markets. National institutions will need to provide better extension support, agricultural implements and technology to farmers, regulate medium and large agribusinesses and prioritize those areas most threatened and vulnerable in the ‘crescent of deforestation’ in the regions south and east.

The article examines past and current institutional shortcomings in forest governance in Amazonia. Given the demonstrated constraints posed by these arrangements, the paper turns to discuss the adaptive governance framework and how it might inform ecosystem management in Amazonia.

## 2. Institutional shortcomings in the management of Amazonia: past and present

Brazil has experienced a rapid evolution of institutions and instruments to manage Amazonia—from community-based extractive reserves established in the 1990s (Brown & Rosendo 2000; Hall 2004), state taxation schemes (May 2002), green certification of forest products (May 2005; Morsello 2006) and more recently experiments with forest-based carbon trading (May 2002; Boyd *et al.* 2007*a*). So far, national and local strategies to protect Amazonia have been largely ineffective, despite the advancement of a variety of approaches, including moves towards decentralization (e.g. the new Brazilian constitution of 1998) and the shift of power and revenues to state and municipal levels. Four environmental regimes govern Amazonia: state (laws and enforcement); state–community (co-management); state–market (concessions); and market–community (green markets). These modes of governance are briefly described and their shortcomings are examined.

Government failure to protect Amazonia has at the core been its development policy over the past 40 years. The primary policy objective has been to integrate Amazonia into the national economy. This has led to widespread and intensive logging, large-scale mechanized soya bean production, credit subsidies for large and small farmers (although the former is in decline), transportation investments and rapidly growing urban areas (Kaimowitz 2002). The federal 2000–2003 development plans for a ‘Forward Brazil’ (Avanca Brazil) comprised US$40 billion planned infrastructure and energy projects for the Amazon region (Kirby *et al.* 2006), despite knowledge that land concentration generally occurs on the edges of frontiers opened up by corporate interests such as mining and logging, and that migrant farmers tend to pivot towards markets and roads (Kaimowitz 2002, p. 228). Roads are one of the major contributors to deforestation. Over 80% of deforestation in the Amazon of Brazil (1991–1994) took place within 50 km of four main road networks (Lele *et al.* 2000). More recently, Kirby *et al.* (2006) confirm that both paved and unpaved roads are consistently important drivers of the process of deforestation. They emphasize that without road access, colonization and deforestation are virtually absent (p. 443). This suggests that with policies such as Avanca Brazil and the increasing pressure of migration by poor landless labourers (23 million in 2003 cited in Kirby *et al.* 2006), the available institutional mechanisms are not sufficient to counteract the drivers of deforestation.

Attempts to protect forest on public lands include national forest, extractive reserves and sustainable development reserves. In 2004, 32% of Brazilian Amazon forest (total 4.1 million km^2^) was located within protected areas (PAs), primarily indigenous lands (940 000 km^2^; 23%). Brazil is implementing a major extension of PAs by 2009, with 270 000 km^2^ of strict PAs, and 500 000 km^2^ of sustainable production forest (Amazon Region Protected Areas programme, National Forest Programme; Malhi *et al.* 2007). In practice, many protected areas and indigenous territories exist only on paper and little is known as to what will happen if transport access improves and pressures are brought on these parks in the process (Kaimowitz 2002). Despite the adverse impacts of the 2005 crises on the state of Acre, it is reputed to have a successful conservation-oriented approach to development. Kirby *et al.* (2006) attribute this success to its history of commercial extractivism, institutional capacity and low pressure on state forests up to 1992.

State–community arrangements are otherwise known as co-management regimes or partnerships between the state and the community institutions (Berkes 2002). Co-management is based on the assumption that conservation is more likely to succeed with the participation of local communities. One such co-management regime in Brazil is the extractive reserve. Extractive reserves were established in the 1990s, created as innovative and new mechanisms to provide land rights to rubber tapper associations. Acre, for example, is a state that is experimenting with ‘neoextractivism’ of forest goods and service where it is helping to aid production and marketing of forest products (Krainer *et al.* 2003). Some suggest that extractive reserves contribute significantly to conservation and development (Ruiz Perez *et al.* 2005), while others critique them. For instance, extractive reserves have been politically empowering to communities, but not financially rewarding (Brown & Rosendo 2000). Institutional capacity is often weak, thus making the practicalities of gearing socially and politically complex organizations towards single goals difficult (Hall 2004). The time horizon for transformation is often underestimated and community organizations have been created without adequate planning and forethought. Property rights regimes are also considered too stagnant in extractive reserves (Goeschl & Camargo Igliori 2006). In a nutshell, extractive reserves are unable to compete with agriculture, cattle ranching and new product lines. As noted by Southgate (1998), the harvesting of non-timber forest products is seldom financially rewarding since markets are scarce and alternative land uses more profitable.

Many of the regions other co-management examples, e.g. marine extractive reserves, encounter similar challenges. The maritime and fisheries reserves are often administered by top-down structures and experience administrative mismanagement and local conflicts (Berkes & Seixas 2004). Moreover, monitoring of resources is often absent and gaps exist in the forms of knowledge possessed by government experts and local counterparts (Seixas 2004). Other state–community initiatives such as ecotourism require high levels of amenities, thus constraining the number of visits and many of the financial returns going to urban firms (Keipi 1999; Kaimowitz 2002).

Public arrangements include Brazil's novel concession policy—the National Forest Programme (Flonas), in which public lands are set aside for permanent production forests where loggers can harvest timber (Rocha *et al.* 2004). Flonas is an innovative forest concession system, which aims to stabilize the existing exploitative logging concessions based on a system of bidding for concessions, which to win also takes into account the bid price, proposed management plan and credibility of the bidder. By 2010, the Brazilian government plans to establish 50 Mha of national forests in the Amazon (Verissimo & Cochrane 2003). More than 1 Mha of managed forests exist in the Amazon (Verissimo & Cochrane 2003), yet competition between sustainably managed and low-cost illegal logging remains a matter of serious concern.

While the state has an important role to play in promoting this system, public forestry institutions remain under-resourced in Brazil to deal with the many drivers of land-use change (Lele *et al.* 2000). For Flonas to function, significant institutional capacity is needed, which requires that institutions are better equipped to respond to surprise, such as dieback and larger administrative autonomy to respond simultaneously to market and forestry management demands (Paladino Correa de Lima & McDaniel 2002).

Market–community arrangements have emerged in more recent years with a focus on environmental services, such as non-timber forest products and carbon sequestration under the Clean Development Mechanism of the Kyoto Protocol. These arrangements aim to put an economic value on resources by transferring the externalities and social costs in the price of goods and services to the consumer. In Brazil, these arrangements include forest carbon (May *et al.* 2004; Boyd *et al.* 2007*a*), forest certification (May 2005), non-timber forest products (Morsello 2006) and fair-trade organic products. Understanding the impacts of these arrangements remains limited. However, recent evidence suggests that market–community arrangements are not necessarily able to raise people out of poverty, aside from situations of severe income scarcity (Morsello 2006). Market–community arrangements can also incur trade-offs between increased production of one commodity (e.g. Brazil nut or carbon) and food security and, as in the case of Brazil nut production, actually ‘threaten food security of communities lacking regular access to purchased food’ (Morsello 2006, p. 489). Carbon forest mitigation schemes based in remote areas could potentially incur similar trade-offs between labour for food production and cultivation of carbon trees for cash. The specific impacts of carbon forest mitigation in Brazil have, so far, been constrained by the lack of institutional capacity to guide, monitor and implement projects successfully (Boyd *et al.* 2007*a*), while similarly, payments for ecosystems often experience problems with carbon measurements, high transaction costs and equity issues. Market–community arrangements may also experience gender-related consequences. Examples of this include the restriction of women's access to e.g. Brazil nut income (Morsello 2006), tree planting benefits or training in forest management (Boyd 2002). Other common challenges include low market prices for products, lack of implementation capacity, exacerbation of existing community inequalities and limited room for negotiation between market and community actors. Engaging communities in these arrangements, to protect forests, requires balancing stringent standards and flexibility, as illustrated by the experience of forest certification in Brazil. May (2005) suggests that the rampant illegal deforestation in Amazonia has limited the impact of certification and calls for more flexible standards in order to realistically compete with the informal trade in forest products.

## 3. Thinking out of the box: adaptive governance as a lens for future transformations

One of the noticeable shortcomings in the management of Amazonia is that each combination of arrangements mentioned is hampered by some kind of structural barrier. Moreover, the relationship between the structures and the biophysical system seem to be overlooked. In searching for ways to better understand the changes that are ongoing, i.e. complex and mega environmental problems, conventional steady-state and linear models of ecological and social change lend insufficient understanding. The adaptive governance framework, although by no means a panacea, seems to capture the importance of adaptive and transformative capacity as a prerequisite for addressing uncertainty and change in systems (Folke *et al.* 2005). This includes identifying dynamic interaction among key individuals, social networks, institutions and ecological systems, underpinned by good science and local knowledge.

Adaptive governance is a lens that can help to identify both positive resilience (strengths) and vulnerability (weaknesses) in the system, i.e. mechanisms that lead to strengthening management or key institutions that sabotage processes. Positive resilience is the ability to buffer, reorganize and learn from crisis. As a framework, adaptive governance explicitly considers social and ecological systems as linked—rather than as separate entities (Folke 2007). Adaptive governance looks for identifying nodes within a network so that resources can be channelled and the ways by which incremental change can take place. It focuses on reflexivity and learning by doing, on the most vulnerable systems that include both human and ecological systems and on the forms of collaboration or partnerships, knowledge, social learning and forms of engagement. The four basic conditions that underpin adaptive governance include: to build knowledge and understand resource and ecosystem dynamics, which requires incentives and human capacity to monitor and translate signals; to feed ecological knowledge into adaptive management processes, whereby successful management includes continuous testing, monitoring, re-evaluation rather than optimizing based on past records; to accept uncertainty and be prepared for change and surprise, i.e. institutions are prepared for both ecosystem management changes and unpredictable changes such as climate change (e.g. storms, hurricanes, pests, disease outbreaks); and to support flexible institutions and multilevel governance systems through networks, operationalized through adaptive co-management, which is adaptive management with multiple level linkages and bridging organizations.

### (a) Knowledge needs, networks and hybrid institutions in Amazonia

A general constraint for forest management strategies in Amazonia is the lack of knowledge and data on the ecological system (du Toit *et al.* 2004). Yet, Brazil's strengths perhaps lie in its successful network of experts, illustrated in the 2005 crisis, which were available to freely exchange information, the information that also became more widely available via the internet to local political figures, the media, professionals and leaders of civil society groups helping to support extensive bottom-up initiatives. This experience suggests that there are already networks in place and social capital available to tap into in designing future management plans for Amazonia. Although there still remains a lack of data on dieback, models, physiological information, reliability of data and ideas of where to prioritize efforts to collect data, the capacity and networks are available to start collating important data, such as that on forest baselines which is also often absent or scattered. A priority for the government of Brazil could be to focus on building on existing scientific initiatives. One such effort is the Amazon Forest Inventory Network (RAINFOR)—an international network of inventory plots established across the Amazon for long-term monitoring of biomass and dynamics of Amazonian forests (Malhi & Phillips 2004; Phillips *et al.* 2005; Peacock *et al.* 2007)—set up as part of Carbonsink, the European contribution to the large-scale biosphere–atmosphere experiment in Amazonia (LBA) (http://www.geog.leeds.ac.uk/projects/rainfor/index.html). The LBA, an evident example of ongoing efforts to construct a better understanding of the Amazon's ecosystems, is a 10-year international research collaboration (1995–2005) led by Brazil. The research component of the LBA, LBA-Eco, focuses on synthesizing knowledge on carbon dynamics, sustainability, system functioning, hydrometeorology and future projections for the Amazon. The next step will be to consider how to feed ecological knowledge into adaptive management processes by continuous testing, monitoring and re-evaluation of data. This condition relates to a wider discussion about the need for change from the current preservation-oriented biological approach to a dynamic one that captures ‘nature under change’ (Folke *et al.* 2004) and emphasizes the importance of local knowledge—the consequences of such a change have yet to be analysed explicitly for Amazonia. From §3 it would appear, however, that there still remain barriers to the integration of local knowledge in adaptive management processes in Amazonia, as well as lack of institutional capacity for monitoring and evaluation. It might be worthwhile, for example, examining how remote and rural communities are gaining access to web-based portals such as that used by the LBA-Eco community.

In dealing with ecological surprise with severe consequences, it may also be necessary to move from precautionary governance to a complexity principle (Boyd *et al.* 2007*b*), thereby accepting uncertainty and preparing for change. The 2005 dieback crises resulted in important lessons with regards to the need for state-of-the-art technology and local observations in monitoring the situation (Brown *et al.* 2006). On reflection, it will be important to continue to build human capacity to anticipate and prepare for future such events. In Amazonia, a better understanding of coupled ecological and social system is called for. This will require continued effort by natural and social scientists to explore collaboratively how society can better anticipate system change and its consequences.

The governance arrangements presented in §3 are largely structural along hierarchical lines and, as such, appear vulnerable to abrupt ecological change. However, promising examples of new multi-actor arrangements are springing up in Amazonia. These so-called ‘hybrid’ institutions comprise state, private *and* community initiatives (Agrawal & Lemos 2007). In Mato Grosso, for example, 17 IPAM employees are attempting to understand how cattle ranchers and soya farmers are able to take better care of their forests and land while still making profits. In this initiative, IPAM has enabled private land holders to sign up to a ‘registry of socioenvironmental responsibility’ (Cadastro de compromisso socioambiental), which it created jointly with Aliance da Terra, a non-governmental organization (NGO) of ranchers and soya farmers, headed by a visionary land owner and rancher (D. Nepstad 2007, personal communication in Butler 2007). Another example is the Amazon Soya Moratorium, issued in July 2006, initiated by Greenpeace and driven forward by the vegetable oil industry with the aim to ensure that industry avoids purchasing soya from recently cleared land for 2 years (Butler 2007). Another example is in northwest Mato Grosso where the aligned efforts of socio-environmental organizations aim to build synergy among their respective achievements and prolong the effects of each individual organization's activity (May *et al.* 2004). Several federal and local government organizations and NGOs have built a cooperative network among their respective actions in the region, including a World Bank Global Environment Facility (GEF)-funded project to ‘Promote Conservation and Sustainable use of Biodiversity’ implemented by Pro-Natura, the Peugeot funded Carbon Sequestration Project, the Italian funded ‘Fire Combat Programme’ administered by the Centro de Vida Institute and the Group of Seven financed Integrated Environmental Management Programme carried out by the state environmental agency FEMA, among other initiatives underway (May *et al.* 2004).

Adaptive governance lends further ideas on how financing mechanisms, such as carbon markets, can contribute more broadly to sustainable development, and there are suggested ways in which the design and implementation of markets could provide benefits to low-income communities (Skutsch 2005; Boyd *et al.* 2007*c*). For instance, finances for carbon forestry could be allocated to collate data and be coordinated with remote-sensing facilities. If on a scale large enough, it may be feasible to bundle a large proportion of areas. In this process, it will also be necessary to have an understanding of the social networks and organizational representatives, including local and community actors, and to whom to channel payments. The type of information required for assessing the carbon value of forests could also pay for other types of monitoring of biodiversity and socioeconomic impacts of carbon initiatives.

## 4. Conclusion

One of the major environmental challenges of the twenty-first century is the continued rapid deforestation of Amazonia. The 2005 events in Amazonia emphasize the unprecedented challenges facing the people and ecosystems of Brazil. The examination of ecosystem management institutions in Amazonia points to structural barriers across all forms of institutional arrangements. In discussing adaptive governance, it appears that Brazil has already established important networks of knowledge and a number of novel initiatives that are emerging in response to past management failures. These new arrangements are novel in the way that they seem to have evolved to navigate structures and insurmountable drivers of deforestation. The federal government will have to think carefully about how to support and enhance these initiatives and knowledge networks. To conclude, saving Amazonia will require a great deal of effort across all scales of administration and decision-making; however, the networks of knowledge and novel use of technology such as satellite imagery are encouraging examples of action. Perhaps adaptive governance can lend insights into how to extend institutions beyond structures to enhance agency of groups and individuals at many different scales.
